# Honokiol Antagonizes Cadmium-Induced Nephrotoxicity in Quail by Alleviating Autophagy Dysfunction, Apoptosis and Mitochondrial UPR Inhibition with Its Antioxidant Properties

**DOI:** 10.3390/life12101574

**Published:** 2022-10-10

**Authors:** Kanglei Zhang, Wenxuan Dong, Jiahui Li, Zhonggui Gong, Wenjing Liu, Shuangjiang He, Hui Zou, Ruilong Song, Gang Liu, Zongping Liu

**Affiliations:** 1 College of Veterinary Medicine, Yangzhou University, 12 East Wenhui Road, Yangzhou 225009, China; 2 Jiangsu Co-Innovation Center for Prevention and Control of Important Animal Infectious Diseases and Zoonoses, Yangzhou 225009, China; 3 Joint International Research Laboratory of Agriculture and Agri-Product Safety, The Ministry of Education of China, Yangzhou University, Yangzhou 225009, China

**Keywords:** Honokiol, cadmium, kidney, autophagy, apoptosis, UPR^mt^

## Abstract

Japanese quail is a highly economically valuable bird due to its commercial production for meat and eggs. Although studies have reported Cadmium (Cd) is a ubiquitous heavy metal that can cause injury to various organs, the molecular mechanisms of Cd on quail kidney injury remain largely unknown. It has been reported that Honokiol (HKL), a highly functional antioxidant, can protect cells against oxidative stress effectively. This study was conducted to investigate the effects of Cd on quail kidneys injury and the protective effect of HKL on Cd-induced nephrotoxicity. A total of 40 Japanese quails were randomly divided into four groups: the control group, Cd treatment group, Co-treatment group and HKL treatment group. The results showed that Cd resulted in significant changes in growth performance, kidney histopathology and kidney biochemical status, antioxidant enzymes and oxidative stress parameters, and ultrastructure of renal tubular epithelial cells, compared with controls. Cd increased the expression of autophagy-related and apoptosis-related genes, but decreased expression of lysosomal function-related and UPR^mt^-related genes. The co-treatment group ameliorated Cd-induced nephrotoxicity by alleviating oxidative stress, inhibiting apoptosis, repairing autophagy dysfunction and UPR^mt^ disorder. In conclusion, dietary supplementation of HKL showed beneficial effects on Japanese quail kidney injury caused by Cd.

## 1. Introduction

Cadmium (Cd) is a highly toxic heavy metal pollutant, that is widely distributed in the biosphere related to various human activities [1]. Cd pollution has occurred frequently in recent years, and has caused serious harm to livestock production and human public health [2,3,4,5]. Absorbed Cd is extremely difficult to remove from the body and has a long biological half-life of 15–30 years [6]. Long-term exposure to Cd is related to the occurrence and development of many diseases, such as hypertension, osteoporosis, diabetes, renal fibrosis, and interstitial renal nephritis [7,8]. The kidney is the main organ for Cd accumulation and damage, and the damage caused by Cd is mainly manifested as acute kidney injury and chronic kidney disease, which is related to lesions of parenchymal cells such as renal tubular cells and mesangial cells [9,10]. The contents of blood urea nitrogen (BUN), creatinine (Crea) and uric acid (UA) are significantly increased, the expression of kidney injury molecule-1 (Kim-1) increased, and the proximal tubule cells were subjected to edema and hypertrophy in Cd-induced nephrotoxicity of rats [11,12]. Japanese quail meat has the characteristics of high protein content, low fat content, low calorific value, and high vitamin A content, which makes the quail meat very popular among consumers [13]. Therefore, quail were used as the experimental subject to investigate the effect of Cd on nephrotoxicity in the present study.

Although Cd is not a redox active metal, it causes cellular oxidative stress by inducing an imbalance in the oxidative-antioxidant system. Mitochondria are the main sites of reactive oxygen species (ROS) production and also the target organelles for Cd-induced cytotoxicity, which ultimately results in increased ROS production during Cd exposure. It has been reported that the thiol protein is very important in cellular antioxidant defense and redox signaling, and the combination of Cd and thiol inhibits the function of the antioxidant defense system, which further aggravate oxidative damage [14]. In vivo and in vitro studies have found that Cd induces mitochondrial damage, increased content of lipid peroxide products, decreased antioxidant enzyme activity or antioxidant content, increased nuclear translocation of nuclear factor (erythroid-derived 2)-like 2 (Nrf2) and elevated target protein expression, and altered trace element content in vertebrate renal tubular epithelial cells [15,16,17,18].

Autophagy is a highly conserved lysosome-dependent cycling process in eukaryotic cells, and plays a vital role in the survival, differentiation, development and homeostasis of cells through the degradation of organelles, proteins and macromolecules in the cytoplasm, as well as reuse of degradation products [19]. Similarly, apoptosis is a programmed cell death process with typical morphological characteristics including plasma membrane blistering, cell shrinkage, chromatin condensation and fragmentation. Numerous studies have found that autophagy and apoptosis are involved in Cd-induced nephrotoxicity in mammals [20,21,22,23]. Notably, studies have reported that autophagy caused by Cd exerts a protective effect on renal tubular damage in the initial stage, while continuous Cd exposure induces lysosomal alkalization that blocks autophagy flux and cargo degradation, causing renal tubular cell damage and apoptosis [20,21,24]. In addition, many studies have found Cd up-regulates the expression of autophagy-related genes including *Atg5*, *Lc3b*, *Becn1,* and apoptosis-related genes including *Casp-3*, *Bak-1*, *Bax*, increases the number of autophagosomes, and induces apoptosis in duck kidneys and renal tubular epithelial cells [16,25,26]. The mitochondrial unfolded protein response (UPR^mt^), an important molecular activity in mitochondrial quality control, is induced by the accumulation of large numbers of denatured or misfolded proteins within the mitochondria and exceeds their clean-up capacity, thereby regulating mitochondrial quality and function [27]. Silent mating type information regulation 2 homolog-1 (SIRT1) and silent mating type information regulation 2 homolog-3 (SIRT3) are the main coordinators of the UPR^mt^ and activate the target protein peroxisome proliferators-activated receptor γ coactivator 1alpha (PGC-1α) and peroxisome proliferators-activated receptor γ coactivator 1 beta (PGC-1β) through their deacetylase activity [28,29]. Nuclear factor (erythroid-derived 2)-like 1 (NRF1) and its target gene mitochondrial transcription factor A (TFAM), as transcription factors in the nucleus and mitochondria, respectively, play an important role in regulating the transcription of genes related to mitochondrial biogenesis and function [30,31]. However, it remains largely unknown whether Cd contributes to quail kidney injury by affecting autophagy, apoptosis, and UPR^mt^, which requires further study.

Honokiol (HKL) is a compound extracted from the bark of Chinese herbal medicine Magnolia officinalis. It has a variety of biological and pharmacological properties, including anti-oxidation, anti-inflammatory, anti-apoptosis, and lowering blood sugar effects [32,33]. The protective effect of HKL against cisplatin-induced renal injury may be mediated by reducing ROS production, inhibiting caspase-3 expression and repairing mitochondrial membrane potential collapse [34]. The administration of HKL can maintain blood glucose control and prevent or delay the development of diabetic nephropathy in type 2 diabetic mice [35]. In addition, HKL significantly alleviates the increase of renal BUN and Crea, histological damage and apoptosis caused by ischemia-reperfusion (I/R) [36,37]. Therefore, this study used quail as an animal model to explore the mechanism of Cd-induced nephrotoxicity in poultry, and provides potential applications for HKL to prevent kidney injure in breeding.

## 2. Materials and Methods

### 2.1. Animal Experiment and Chemicals

The animals used in this experiment were 1-day-old Japanese quails (40 birds, and were purchased from a farm in Zhenjiang City, Jiangsu Province). After pre-feeding for 1 week, they were randomly divided into the following four groups: control group (only normal feed), Cd treatment Group (normal feed containing 75 mg/kg CdCl_2_), Cd and HKL co-treatment group (normal feed containing 50 mg/kg HKL pre-fed for 4 weeks, then supplemented with 75 mg/kg CdCl_2_ to continue feeding 4 weeks), HKL group (normal feed containing 50 mg/kg HKL). The temperature, and humidity conditions, and light time in the breeding environment were in line with the animal breeding requirements of the Animal Management Committee of Yangzhou University. From 12 h before the end of the animal experiment, all animals were fasted and then dissected in accordance with the protocol and ethical procedures. The study was approved by the Animal Care and Use Committee of Yangzhou University (Approval ID: 202012-201). Blood samples were allowed to stand at 37 °C for 30 min then centrifuged at 2500× *g* rpm for 10 min and the supernatant was collected. Part of the kidney tissue was immersed in neutral tissue fixative and 2.5% glutaraldehyde fixative, and the remaining tissue was stored in a refrigerator at −80 °C for detection of related indicators.

CdCl_2_ was obtained from Sigma-Aldrich (St. Louis, MO, USA), HKL and purchased from Shanghai Yuanye Bio-Technology Co., Ltd. (Shanghai, China).

### 2.2. Detection of Biochemical Indicators in Serum

The main biochemical indicators of renal function were measured in quail serum using AU5800 automatic blood biochemical analyzer (Beckman Coulter, Brea, CA, USA), including Crea, uric acid (UA) and blood glucose.

### 2.3. Preparation and Observation of Transmission Electron Microscope Samples

Kidneys were divided into small pieces, fixed in buffered glutaraldehyde 2.5% at 4 °C for 2 h, then the samples were post-fixed in 1% osmium tetraoxide for 2 h at 4 °C and dehydrated in different concentrations of ethanol. The samples were immersed in a mixture of equal volumes of ethanol (100%) and acetone (100%) for 15 min, then in acetone (100%) for another 15 min. Subsequently, the samples were placed in equal volumes of epon and acetone for 60 min, then 1:2 acetone: epon for 60 min. After that, the samples were immersed in epon only in embedding capsules and placed in a 60 °C oven overnight for polymerization. Ultrathin sections (60 nm) were prepared. The sections were subjected to double staining with 2% uranyl acetate for 10 min followed by Reynold’s lead citrate solution for 10 min. The morphology of the cell nucleus, the ultrastructure of mitochondria and autophagosomes were observed and photographed using a transmission electron microscope (Philips, AMS, NL) under different magnifications.

### 2.4. RNA Isolation and Quantitative Real-Time PCR

According to whole genome sequence of the studied gene in Gen Bank, Primer 5.0 software (Premier, Palo Alto, CA, USA) was used to design primers for related genes. The designed primers were handed over to the Invitrogen company for synthesis. The primer sequence is shown in Supplementary Table S1. RNA was separated using Trizol (Ambion, UT, Austin, TX, USA). RNA was subsequently reverse transcribed into cDNA using PrimeScript™ RT Master Mix (Takara, Japan). Quantitative PCR was performed using SYBR Green™ Premix Ex Taq™ (Takara, Japan). QRT-PCR was performed using a two-step method, and the reaction conditions were set at 95 °C, 30 s; 60 °C, 37 s; 40 cycles. All reactions were carried out using the 7500 Real Time PCR System (Applied Biosystems, Shanghai, China). After the reaction was finished, the specificity of the PCR product was determined by a melting curve. *Gapdh* was used as the internal reference, and the relative fold change was calculated by the comparative CT method.

### 2.5. Determination of Elements

About 250 mg of fresh kidney tissues were weighed and subsequently placed in an oven for 48 h to completely dehydrate to obtain dry tissue. The dry tissues were accurately weighed using an electronic analytical balance. Each dry tissue sample was dissolved with 3 mL HNO_3_ (excellent grade pure) and placed in a microwave digester (Intertek, London, UK) for tissue digestion, and a blank sample tube was set with only 3 mL HNO_3_ added. After dissolution the solution was transferred to a constant volume tube, and ultrapure water was added to a constant volume of 10 mL and mixed. The content of Cd and other trace elements were measured using an atomic absorption spectrophotometer (PerkinElmer, Waltham, MA, USA), and blank sample tube was used as a zero to remove background interference. The final element contents are presented as the ratio of the detected element content (μg or mg) to the dry weight (g) of the tissue sample.

### 2.6. Hematoxylin and Eosin (H&E) Staining

Freshly separated quail kidney tissue was fixed in neutral fixative for 24 h. The sample was rinsed with running water for 12–24 h. The samples were placed in a series of ethanol with different concentrations of 15, 30, 50, 70, 80, 95 and 100% for 45 min to 1 h to completely dehydrate the tissue. The tissue was first placed in an equal volume mixture of absolute ethanol and xylene for 60 min, and then placed in xylene I and Ⅱ for 60 min. The samples were embedded in paraffin and formed into paraffin sections by a sectioning mechanism. The slices were placed in xylene I and Ⅱ for 10 min each. The slices were further placed in the mixture of xylene and absolute ethanol for 2 min. The samples were placed in 100, 90, 80 and 70% alcohol for 5 min and rinsed with running water. Hematoxylin staining was conducted for 5–10 min, and the samples rinsed with running water. The samples were treated with 0.5% hydrochloric acid ethanol for 1–3 s and washed with running water, then stained with 0.5% eosin solution for 1–3 min and rinsed with running water. The slices were placed in 70, 80, 90, 100% alcohol for 10 s, and then in xylene 2 min. The slices were sealed with neutral gum, and then histopathological changes were observed under an optical microscope.

### 2.7. Apoptosis Detection by TUNEL Assay

Apoptosis was assessed in kidney tissues using the terminal deoxynucleotidyl transferase-mediated nick-end labeling (TUNEL) of fragmented nuclei assay. According to the manufacturer’s instructions (Roche, Mannheim, Germany), the paraffin-embedded sections of kidney tissues of different groups were processed. Finally, apoptotic cells within the kidney tissues were detected with a fluorescent microscope. Apoptotic cell number in each section was calculated by counting the number of TUNEL-positive apoptotic cells in four fields per slide randomly at 400× magnification.

### 2.8. Detection of MDA and Antioxidant Enzymes Activity

Kidney tissue (80–100 mg) was weighed and rinsed with 1 mL of normal saline, followed by centrifugation at 2500× *g* rpm for 5 min and the supernatant was removed. The tissue pellet was homogenized with 1 mL of normal saline to produce a 10% tissue homogenate. Then, the homogenate was centrifuged at 2500× *g* rpm for 10 min and the supernatant was collected. The level of malondialdehyde (MDA) and glutathione (GSH), and the activities of catalase (CAT), total superoxide dismutase (T-SOD), and total antioxidant capacity (T-AOC) were evaluated by commercial kits according to the manufacturers’ protocols. The protein concentration of the samples was determined by bicinchoninic acid (BCA) assay to normalize the level. All detection kits used were purchased from Nanjing Jiancheng Bioengineering Institute (Nanjing, China).

### 2.9. Western Blot

Total protein from kidney tissue was extracted by a commercial kit (NCM, Suzhou, China) and protein concentration was determined using a BCA protein detection kit (Beyotime, Shanghai, China). Protein samples were separated by SDS-PAGE and transferred to polyvinylidene difluoride (PVDF) membranes (Merck, Cork, Ireland). After blocking with TBST containing 5% fat-free milk, PVDF membranes were co-incubated with diluted anti-BAX (ab32503, abcom, Cambs, UK), anti-BCL2 (ab196495, abcom, Cambs, UK), anti-LC3 (L7543, Sigma, St. Louis, MO, USA), anti-SQSTM1 (P0067, Sigma, St. Louis, MO, USA) and anti-β-actin (4970L, CST, Danvers, MA, USA) at 4 °C overnight. PVDF membranes were incubated with secondary antibodies (CST, MA, USA) at 37 °C for around 2 h and washed with PBST. By using the ECL kit (NCM, Shanghai, China), the immune complex was visualized by a Tanon chemiluminescence imaging analysis system (Tanon, Shanghai, China). Densitometry analysis was quantified by Image J software (NIH, Bethesda, MD, USA).

### 2.10. Statistical Analysis

The statistical results are presented in Mean ± SEM (*n* ≥ 3). IBM SPSS Statistics 19 statistical software (IBM, Armonk, NY, USA) was used to perform one-way ANOVA statistical analysis on the data. *p* > 0.05 means that there is no significant difference between different groups; *p* < 0.05 means that the difference between different groups is significant; *p* < 0.01 means that the difference between different groups is extremely significant. GraphPad Prism software (San Diego, CA, USA) was used to draw charts.

## 3. Results

### 3.1. Honokiol Relieves Cd-Induced Quail Kidney Injury

**Figure 1:**
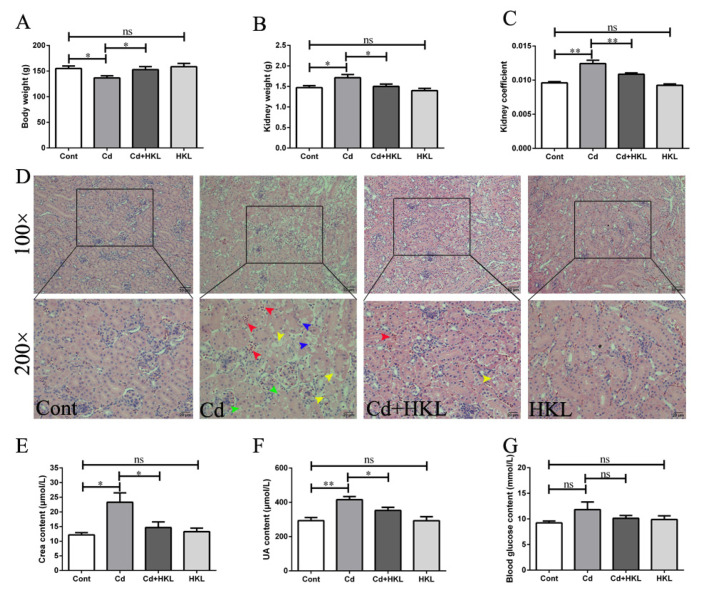
Honokiol alleviates Cd-induced kidney injury in quail. Quail body weight (**A**), kidney weight (**B**) and kidney coefficient (**C**) were analyzed. (**D**) The histological structure of quail kidney was observed by H&E staining. The magnifications were 100 and 200 respectively, and the scale bars indicate 50 and 20 μm respectively. The contents of Crea (**E**), UA (**F**) and Glucose (**G**) in serum were detected. Each experiment was duplicated at least three times (ns, not significant; * *p* < 0.05, ** *p* < 0.01).

To study the effect of HKL on Cd-induced nephrotoxicity, body weight and kidney weight were analyzed first. The results showed that Cd significantly reduced the body weight, and significantly increased the kidney weight and the kidney coefficient compared to control group. However, the co-treatment group had significant reversal of these changes induced by Cd exposure (Figure 1A–C). Histopathological changes were observed to assess the injure of quail kidneys. The renal tubular epithelial cells and glomerular cells in the control group and the HKL group were arranged regularly without any visible changes. The Cd-treated group showed significant damage to morphology, mainly manifested as disordered arrangement (yellow arrow), vacuolization, swelling (green arrow), or even sloughed off (blue arrow) of renal tubular epithelial cells, as well as red blood cells infiltrating the renal interstitium (red arrow). These pathological changes induced by Cd were alleviated significantly in the co-treatment group (Figure 1D). Additionally, renal function-related biochemical indicators showed that in addition to blood glucose, the UA and Crea levels in the Cd-exposed group significantly increased compared to the control group, but the changes caused by the Cd were reversed in the co-treatment group (Figure 1E–G). These results indicate that HKL can effectively alleviate quail kidney damage caused by Cd treatment.

### 3.2. Honokiol Relieves Cd-Induced Oxidative Stress in Quail Kidneys

**Figure 2:**
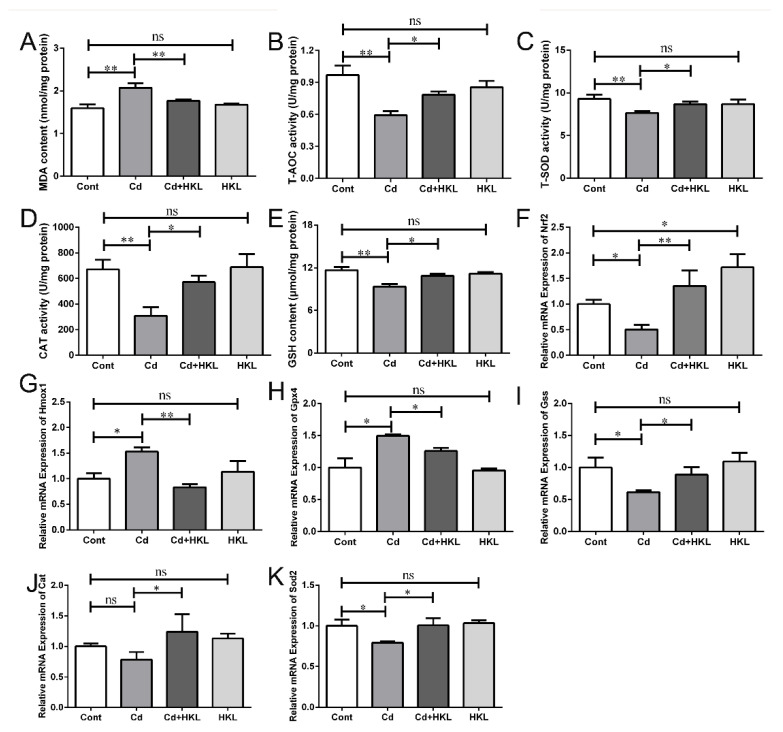
Honokiol repairs Cd-induced oxidative stress in quail kidneys. (**A**) The level of MDA in quail kidney tissues. The activity of T-AOC (**B**), T-SOD (**C**) and CAT (**D**) was detected to evaluate the level of oxidative stress in kidney tissue. (**E**) GSH levels in the kidney tissues of quail. The expression levels of antioxidant-related genes *Nrf2* (**F**) and *Hmox1* (**G**), *Gpx4* (**H**), *Gss* (**I**), *Cat* (**J**) and *Sod2* (**K**) in quail kidney tissues were analyzed using qRT-PCR. The expression of genes was measured by the comparative CT method. Each experiment was duplicated at least three times (ns, not significant; * *p* < 0.05, ** *p* < 0.01).

**Figure 3:**
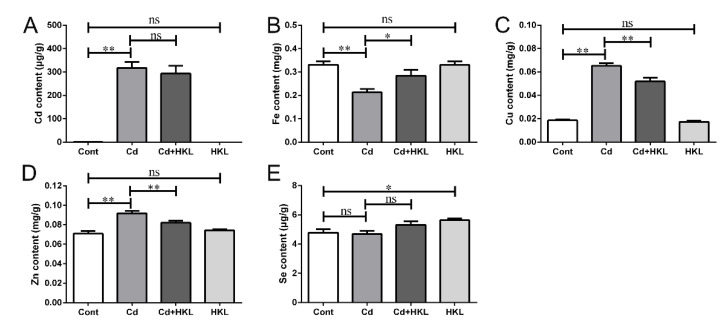
Effects of Cd or Honokiol treatments on the content of Cd and other trace elements in quail kidneys. The contents of Cd (**A**) and trace elements Fe (**B**), Cu (**C**), Zn (**D**) and Se (**E**) in kidneys were detected by atomic spectrophotometer. Each experiment was duplicated at least three times (ns, not significant; * *p* < 0.05, ** *p* < 0.01).

Since oxidative stress is one of the main causes of Cd-induced cytotoxicity, we explored whether the protective effect of HKL on quail nephrotoxicity caused by Cd is related to its antioxidant nature. The oxidation status of the body is coordinated and regulated by the oxidation-antioxidant systems. Among them, the antioxidant system mainly includes a variety of antioxidants and antioxidant enzymes, such as GSH, SOD and so on. First, we detected the content of MDA in quail kidney, which is a product of lipid peroxidation, and its content is widely used as a key indicator to evaluate the oxidation state of the body. The result showed that compared with the control group, Cd significantly increased the MDA content, which was prevented by HKL administration (Figure 2A). Meanwhile, the results of antioxidant enzyme activity and antioxidant content showed that, compared with the control group, T-SOD, T-AOC and CAT activity, and GSH content were significantly reduced in the Cd-treated group, and were alleviated by HKL administration (Figure 2B–E). Subsequently, we detected expression levels of antioxidant-related genes at the transcription level. These results showed that compared with the control group, Cd significantly reduced the gene expression levels of *Nrf2*, *Sod2*, and *Gss,* but significantly increased the expression levels of *Hmox1* and *Gpx4*, which were reversed by HKL administration. However, *Cat* gene expression did not change significantly in the Cd group and the co-treatment group (Figure 2F–K). Finally, trace element contents in the kidney were detected by an atomic absorption spectrometry. The results showed that compared with the control group, Cd caused a significant increase in Zinc (Zn) and Copper (Cu) contents, but a significant decrease in Iron (Fe) content in quail kidney tissue. The co-treatment group had significant reversal of the changes of trace elements caused by Cd. However, there was no significant change in the Selenium (Se) content of the Cd group and the co-treatment group (Figure 3A–E). These results indicate that the protective effect of HKL on quail nephrotoxicity induced by Cd is partially related to its antioxidant properties.

### 3.3. Honokiol Alleviates Cd-Induced Autophagy Dysfunction in Kidney

**Figure 4:**
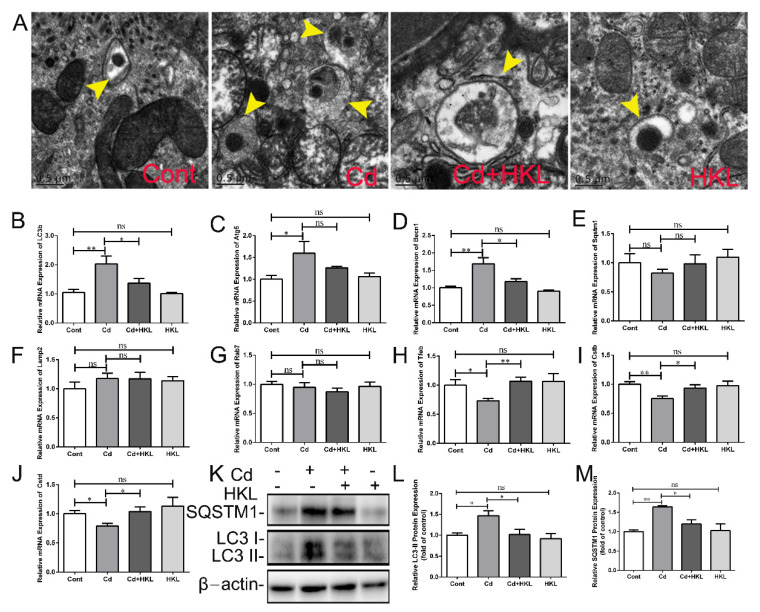
Honokiol alleviates the Cd-induced autophagy blockade and lysosomal dysfunction in quail kidneys. (**A**) Autophagosomes or autolysosomes (the arrows represent) observed by transmission electron microscopy. The magnification is 18,500, and the scale bar is 0.5 μm. Expression levels of autophagy-related genes *Lc3b* (**B**), *Atg5* (**C**), *Becn1* (**D**) and *Sqstm1* (**E**) were analyzed using qRT-PCR. The expression levels of lysosomal function-related genes *Lamp2* (**F**), *Rab7* (**G**), *Tfeb* (**H**), *Cstb* (**I**) and *Cstd* (**J**) were analyzed using qRT-PCR. The expression of genes was measured by the comparative CT method. Representative Western blot images (**K**) and quantitative analysis (**L**,**M**) of SQSTM1 and LC3 protein in total cellular lysates. Each experiment was duplicated at least three times. (ns, not significant; * *p* < 0.05, ** *p* < 0.01).

Autophagy is a strictly conserved metabolic process in evolution. A large number of studies have proved that autophagy is involved in Cd-induced nephrotoxicity, and oxidative stress is also a common cause of autophagy [38]. The above results proved that HKL reduce the body’s oxidative stress through its antioxidant properties. Based on this, we further studied the relationship between autophagy and HKL on Cd-induced nephrotoxicity. Transmission electron microscopy showed that the number of autophagosomes or autophagolysosome increased significantly in the Cd treatment group compared with the control group, but the increase caused by Cd was reduced significantly in the co-treatment group (Figure 4A). Secondly, the expression levels of autophagy-related genes were detected by qRT-PCR. The results showed that compared with the control group, the *Lc3b*, *Atg5* and *Becn1* genes were significantly increased in the Cd-treated group, while the expression levels of *Tfeb*, *Cstb* and *Cstd* were significantly reduced. Compared with the Cd-treated group, the *Lc3b*, *Atg5* and *Becn1* genes in the co-treatment group were reduced significantly, and *Tfeb*, *Cstb* and *Cstd* were significantly increased. However, the expression of *Sqstm1*, *Lamp2* and *Rab7* genes did not change significantly in the Cd-treated group and the co-treatment group (Figure 4B–J). Finally, Western blot results showed that Cd caused increased expression of SQSTM1 and LC3 II proteins, suggesting that autophagy is dysfunctional, possibly due to the inability of SQSTM1 and LC3 II proteins to be degraded by autophagy, leading to their accumulation. As expected, HKL supplementation significantly reduced the up-regulation of SQSTM1 and LC3 II proteins in quail kidneys induced by Cd (Figure 4K–M). These results indicate that HKL can alleviate autophagy dysfunction induced by Cd in quail kidneys.

### 3.4. Honokiol Inhibits Cd-Induced Quail Kidney Apoptosis

**Figure 5:**
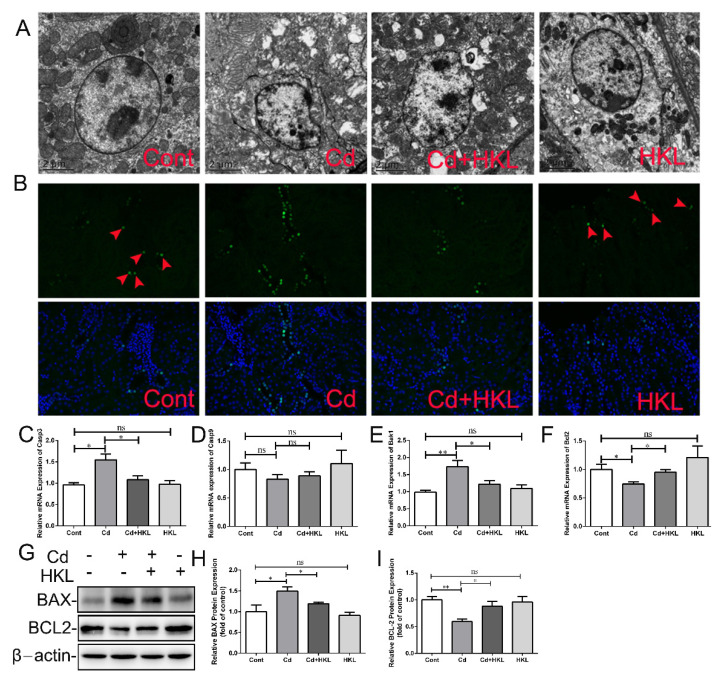
Honokiol inhibits Cd-induced quail kidney apoptosis. (**A**) The nuclear morphology of quail kidneys was observed by transmission electron microscopy. Magnification ×5800, scale bar = 2 μm. (**B**) Tunel staining was used to assess the level of apoptosis in quail kidneys (Arrows indicate Tunel-positive cells). The expression level of the apoptosis-related gene *Casp3* (**C**), *Casp9* (**D**), *Bak1* (**E**) and *Bcl2* (**F**) was detected using qRT-PCR. The expression of genes was measured by the comparative CT method. Western blot images (**G**) and quantitative analysis (**H**,**I**) of BAX and BCL-2 protein in total cellular lysates. Each experiment was duplicated at least three times. (ns, not significant; * *p* < 0.05, ** *p* < 0.01).

Next, we further explored the effect of Cd on apoptosis in the quail kidney and the protective effect of HKL. Transmission electron microscopy was used to observe nuclear morphological changes. The control group and the HKL treatment group presented intact nuclei and homogenized chromatin, but shrunken nuclei were observed in the Cd-treated group. Co-treatment of HKL with Cd significantly alleviated Cd-induced changes in nuclear morphology (Figure 5A). The Tunel staining result showed that the number of Tunel-positive cells was low in the control group and the HKL-treated group, but Cd significantly increased the number of Tunel-positive cells, especially in the renal tubule area. As expected, the number of Tunel-positive cells in the co-treatment group was significantly reduced compared with Cd-treated group (Figure 5B). Subsequently, the expression of apoptosis-related genes was assessed to further prove the effect of co-treatment on the apoptosis of quail kidneys. Compared with the control group, Cd significantly increased the expression of *Casp3* and *Bak1* genes and significantly decreased the expression of the *Bcl2* gene. Compared with the Cd treatment group, the expression of *Casp3* and *Bak1* genes in the co-treatment group was reduced, while the expression of *Bcl2* gene was significantly up-regulated (Figure 5C–F). Finally, Western blot results showed that Cd up-regulated BAX protein expression and down-regulated BCL2 protein expression, while co-treatment of HKL with Cd significantly reversed these changes caused by Cd (Figure 5G–I). These results indicate that HKL plays a protective role in Cd-induced nephrotoxicity by antagonizing apoptosis.

### 3.5. Honokiol Repairs Cd-Induced Inhibition of Mitochondrial Unfolded Protein Response in Quail Kidney

**Figure 6:**
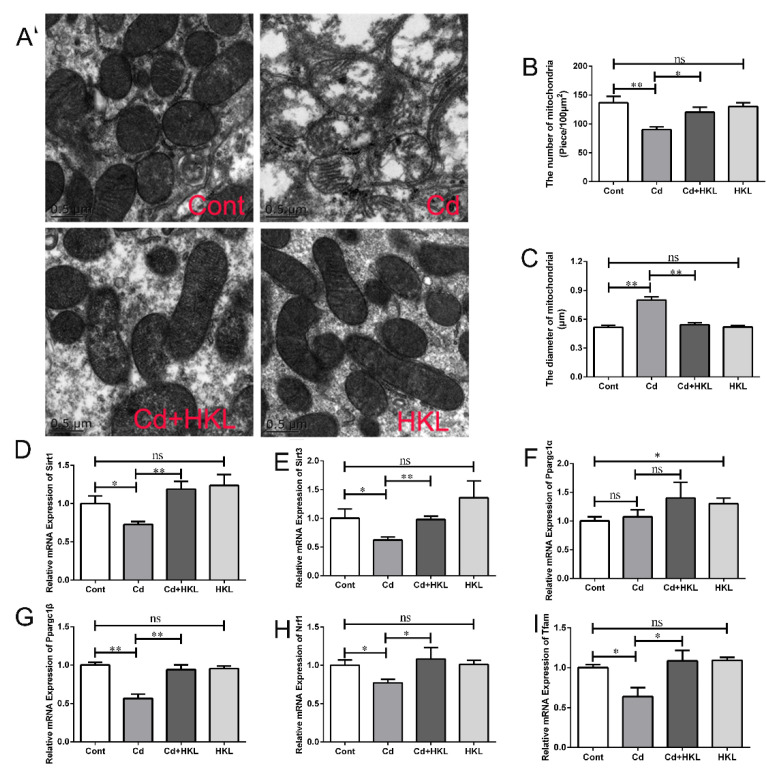
Honokiol attenuates Cd-induced inhibition of the mitochondrial unfolded protein response in quail kidneys. (**A**) Mitochondrial ultrastructure was observed by transmission electron microscopy. Magnification ×18,500; scale bar = 0.5 μm. (**B**) The number of mitochondria was counted. (**C**) The mitochondrial diameter was analyzed. The expression levels of the UPRmt-related gene Sirt1 (**D**), Sirt3 (**E**), Ppargc1α (**F**), Ppargc1β (**G**), Nrf1 (**H**) and Tfam (**I**) were detected using qRT-PCR. Each experiment was duplicated at least three times. (ns, not significant; * *p* < 0.05, ** *p* < 0.01).

Mitochondria are extremely sensitive to Cd toxicity, and mitochondrial dysfunction and biogenesis disorder have been involved in a variety of Cd poisonings. The ultrastructure of mitochondria was observed under transmission electron microscopy. The results showed the mitochondria in kidney tissue of the control group and HKL group showed a complete morphology with regular arrangement of cristae. Compared with the control group, the mitochondrial morphology of the Cd group changed significantly, mainly manifested as mitochondrial swelling and ridge breakage, dissolution or even loss, and mitochondrial membrane destruction. The mitochondrial ultrastructural damage of the quail kidney caused by Cd exposure was significantly improved in the co-treatment group (Figure 6A). In addition, the statistical results of the average diameter and number of mitochondria showed that compared with the control group, Cd significantly increased the average diameter of mitochondria and significantly decreased the number of mitochondria, which were significantly reversed by HKL administration (Figure 6B,C). Next, the expression levels of UPR^mt^-related genes in quail kidney were analyzed. The results showed that compared with the control group, Cd significantly reduced the expression of *Sirt1*, *Sirt3*, *Nrf1*, *Ppargc1β* and *Tfam* genes except the *Ppargc1α* gene, but the down-regulation of these genes caused by Cd in quail kidney tissue was significantly reversed in the co-treatment group (Figure 6D–I). These results indicate that HKL can exert beneficial effects on Cd-induced quail nephrotoxicity by alleviating mitochondrial structural damage, repairing mitochondrial dysgenesis, and maintaining mitochondrial quality control.

## 4. Discussion

Cd is a toxic heavy metal that exists widely in nature and has considerable occupational and environmental hazards. Cd induces the production of ROS and causes oxidative stress, which contribute to the development of various diseases including renal dysfunction, bone disease and cardiovascular disease [2]. Many antioxidants and herbs have been used to counteract the toxicity caused by Cd exposure [39]. HKL is a natural lignan extracted from the Chinese herbal medicine species Magnolia officinalis, with anti-oxidation effects. However, there are few reports on the effect of HKL on Cd toxicity. This study used quail as an animal model to explore the mechanism of Cd on renal injure and the protective effect of HKL on Cd-induced nephrotoxicity. We found that Cd accumulated in quail kidney and caused kidney pathology and functional changes. Secondly, Cd induced oxidative stress, autophagy dysfunction, apoptosis, and UPR^mt^ disorder. Finally, HKL supplementation in the basal diet significantly improved the kidney injury caused by Cd in quail.

Kidney is the main target organ for Cd bioaccumulation, second only to the liver, which makes the kidney exceptionally sensitive to Cd toxicity [40]. Cd causes varying degrees of irreversible kidney damage in poultry, such as chickens, ducks and quails [15,25,41]. Similar to previous results, our study showed that Cd caused body weight loss in quail, but increased kidney weight and the kidney coefficient. Cd also caused pathological changes in kidney tissue, which were mainly manifested as glomerular stenosis, red blood cell infiltration in the interstitium, irregular arrangement of renal tubular epithelial cells, and swelling, and even shedding, of some renal tubular epithelial cells. In addition, Cd increased the UA and Crea content in quail, compared to controls. However, HKL supplementation significantly reversed the Cd-induced changes in the above-mentioned histopathology and renal function-related indicators.

Cd-induced nephrotoxicity is closely related to oxidative stress, resulting in the peroxidation of biological macromolecules, such as proteins, lipids and DNA [14]. Many trace elements are involved in regulating the balance of the oxidative-antioxidant system as key cofactors of antioxidant enzymes or as natural antioxidants; however, Cd can replace or reduce the absorption of trace elements involved in antioxidants [42,43]. Our study showed that Cd caused an increase in MDA content, but a decrease in GSH content, and a decrease in T-AOC, T-SOD and CAT activities in quail kidneys compared to controls. Cd also induced a significant reduction in Fe content and a significant increase in Cd, Zn and Cu content, except for Se content, compared to the control. In addition, Nrf2 is a transcription factor and a key regulator of oxidative stress, which can coordinate the activation of multiple Nrf2-dependent target genes induced by stress. Studies have found that Cd increases the expression of the *Nrf2* gene and its target genes in chicken kidneys [15]. Notably, our study found that Cd significantly altered the expression levels of antioxidant-related genes, except the *Cat* gene, in kidneys, which may be related to excessive Cd bioaccumulation and severe damage in the kidney caused by high concentration Cd exposure in this experiment. HKL supplement partially alleviated the Cd-induced changes in oxidative stress-related indicators in quail kidneys, which may be related to the potent antioxidant properties of HKL [44,45].

Autophagy is an evolutionarily conserved lysosomal-dependent degradation process. Oxidative stress-mediated autophagy dysfunction is beneficial to Cd-induced renal toxicity, which mainly involves the fusion of autophagosomes and lysosomes and lysosomal dysfunction. Previous studies have found that autophagy is involved in Cd-induced duck kidney injury in vivo and in vitro [16,46]. Our study showed that Cd caused the up-regulation of *Lc3b*, *Becn1* and *Atg5* genes and significantly reduced the expression of *Tfeb* and target genes *Cstb* and *Cstd* in quail kidneys. Consistently, transmission electron microscope showed that the number of autophagosomes and autophagolysosomes increased after Cd treatment. HKL supplementation partially reversed Cd-induced changes in renal autophagy-related markers. However, Cd or HKL supplementation had no significant effect on *Sqstm1*, *Rab7a* and *Lamp2* gene expression. Next, we explored the effect of HKL and Cd on apoptosis, i.e., gene-regulated programmed death. Studies have found that Cd induces the expression of apoptosis-related genes or cell nuclear shrinkage in duck kidneys or quail liver [25,41]. In this experiment, Cd caused obvious cell nucleus shrinkage accompanied by crescent-shaped depressions, and increased the number of Tunnel-positive cells significantly. Subsequently, we found that the expression of the pro-apoptotic genes *Casp3* and *Bak1* increased and the expression of the anti-apoptotic gene *Bcl2* decreased after Cd treatment, but the *Casp9* gene did not change significantly compared with the control. In addition, Western blot results showed that Cd increased BAX, but decreased BCL-2 protein expression. HKL supplementation significantly alleviated Cd-induced changes in apoptosis-related markers in quail kidneys. Therefore, dysfunction autophagy caused by Cd may further lead to apoptosis and cause quail kidney damage. In line with with our hypothesis, studies have shown that repairing dysfunctional autophagy can significantly alleviate Cd-induced apoptosis and rat kidney injury [21].

UPR^mt^ is a major stress response and protective mechanism for repairing damaged mitochondria. Studies have found Cd causes the up-regulation of UPR^mt^-related genes in duck kidneys, but the expression of some of them decreased with an increase of Cd concentration [15]. In the present study, Cd caused disruption of mitochondrial structural integrity, loss of cristae, swelling and reduction of mitochondrial numbers. Further, Cd decreased expression of the UPR^mt^-related genes *Sirt1*, *Sirt3*, *Nrf1*, *Ppargc1β* and *Tfam*, except for *Ppargc1α*. HKL supplementation significantly alleviated mitochondrial ultrastructural damage and partially upregulated the downregulation of UPR^mt^-related gene induced by Cd in quail kidneys. We speculate that low-dose Cd compensatively activates UPR^mt^ to eliminate misfolded proteins in the mitochondrion, but UPR^mt^ is eventually inhibited or insufficient with increasing Cd concentrations or prolonged exposure to Cd, resulting in mitochondrial structural damage and dysfunction. In addition, Cd-induced apoptosis in quail kidneys may be related to UPR^mt^ inhibition, because supplementation with HKL reversed the down-regulation of UPR^mt^-related genes induced by Cd, and the level of apoptosis was correspondingly reduced, which may be related to the mitigation of mitochondrial dysfunction by repaired UPR^mt^. Interestingly, further activation of URP^mt^ upregulated the pro-apoptotic gene *Bax* in a study of fat and obesity-associated genes (FTO) on adipocyte metabolism [47]. The difference between the two studies may be due to different cells and different treatments, and the relationship between UPR^mt^ and apoptosis needs to be further investigated. It has been reported that UPR^mt^ and mitophagy, a selective autophagy that degrades damaged mitochondria synergistically, alleviates LPS-induced cardiac dysfunction, and endogenous UPR^mt^ is upregulated and plays a compensatory role in maintaining mitochondrial homeostasis in the context of mitophagy inhibition [48]. Notably, the present results also found that Cd caused an increase in the number of encapsulated mitochondria (Figure 4A, see yellow arrow), accompanied by an increase in the number of autophagosomes or autophagolysosomes. Combined with the Cd-induced decrease in UPR^mt^-related gene expression, our results imply that UPR^mt^ inhibition may play a positive regulatory role in mitophagy activation. Consistent with our results, it has been reported that continued stimulation can cause mitochondrial damage to develop locally in the entire mitochondria, and then activate mitophagy to remove the entire damaged mitochondria, otherwise the dysfunctional mitochondria will cause further cell damage [49].

## 5. Conclusions

Cd causes quail kidney injury by inducing oxidative stress, autophagy dysfunction, UPR^mt^ inhibition and apoptosis, and HKL supplementation significantly alleviates Cd-induced nephrotoxicity, which may be related to the potent antioxidant effect of HKL. In conclusion, this study elaborates the mechanisms underlying nephrotoxicity caused by Cd and provides potential applications for HKL-targeted therapy of kidney injury in livestock and poultry breeding.

## Data Availability

The datasets used and/or analyzed during the current study are available from the corresponding author on reasonable request.

## Supplementary Materials

The following supporting information can be downloaded at: https://www.mdpi.com/article/10.3390/life12101574/s1, Table S1: Primer sequences of mRNA for QRT-PCR.

## References

[1] Järup L. (2003). Hazards of heavy metal contamination. Br. Med. Bull..

[2] Genchi G., Sinicropi M., Lauria G., Carocci A., Catalano A. (2020). The Effects of Cadmium Toxicity. Int. J. Environ. Res. Public Health.

[3] Kar I., Patra A. (2021). Tissue Bioaccumulation and Toxicopathological Effects of Cadmium and Its Dietary Amelioration in Poultry-a Review. Biol. Trace Elem. Res..

[4] Raeeszadeh M., Gravandi H., Akbari A. (2022). Determination of some heavy metals levels in the meat of animal species (sheep, beef, turkey, and ostrich) and carcinogenic health risk assessment in Kurdistan province in the west of Iran. Environ. Sci. Pollut. Res. Int..

[5] Tao C., Zhang B., Wei X., Zhao M., Sun Z., Wang S., Bi J., Qi D., Sun L., Zhang N. (2020). Effects of dietary cadmium supplementation on production performance, cadmium residue in eggs, and hepatic damage in laying hens. Environ. Sci. Pollut. Res. Int..

[6] Bernhoft R.A. (2013). Cadmium toxicity and treatment. Sci. World J..

[7] Nordberg G. (2009). Historical perspectives on cadmium toxicology. Toxicol. Appl. Pharmacol..

[8] Satarug S., Vesey D., Gobe G. (2017). Kidney Cadmium Toxicity, Diabetes and High Blood Pressure: The Perfect Storm. Tohoku J. Exp. Med..

[9] Yang L., Wu K., Chiu W., Wang S., Shih C. (2009). The cadmium-induced death of mesangial cells results in nephrotoxicity. Autophagy.

[10] Wang L., Fan R., Yang D., Zhang D., Wang L. (2019). Puerarin reverses cadmium-induced lysosomal dysfunction in primary rat proximal tubular cells via inhibiting Nrf2 pathway. Biochem. Pharmacol..

[11] Kim K., Lim H., Lim J., Son J., Lee J., Lee B., Chang S., Kim H. (2018). Curcumin ameliorates cadmium-induced nephrotoxicity in Sprague-Dawley rats. Food. Chem. Toxicol..

[12] Hu Z., Zhang H., Yi B., Yang S., Liu J., Hu J., Wang J., Cao K., Zhang W. (2020). VDR activation attenuate cisplatin induced AKI by inhibiting ferroptosis. Cell Death Dis..

[13] Ahmed A., Hamed D., Elsharawy N. (2017). Evaluation of some heavy metals residues in batteries and deep litter rearing systems in Japanese quail meat and offal in Egypt. Vet. World.

[14] Gobe G., Crane D. (2010). Mitochondria, reactive oxygen species and cadmium toxicity in the kidney. Toxicol. Lett..

[15] Sui M., Jiang X., Chen J., Yang H., Zhu Y. (2018). Magnesium isoglycyrrhizinate ameliorates liver fibrosis and hepatic stellate cell activation by regulating ferroptosis signaling pathway. Biomed. Pharmacother..

[16] Zhuang J., Nie G., Yang F., Cao H., Xing C., Dai X., Hu G., Zhang C. (2019). Molybdenum and Cadmium co-induced the levels of autophagy-related genes via adenosine 5’-monophosphate-activated protein kinase/mammalian target of rapamycin signaling pathway in Shaoxing Duck (Anas platyrhyncha) kidney. Poult. Sci..

[17] Wang L., Chen D., Cao J., Liu Z. (2009). Protective effect of N-acetylcysteine on experimental chronic cadmium nephrotoxicity in immature female rats. Hum. Exp. Toxicol..

[18] Wang X., Wang Z., Zhu Y., Zhu S., Fan R., Wang L. (2018). Alleviation of cadmium-induced oxidative stress by trehalose via inhibiting the Nrf2-Keap1 signaling pathway in primary rat proximal tubular cells. J. Biochem Mol. Toxicol..

[19] Parzych K., Klionsky D. (2014). An overview of autophagy: Morphology, mechanism, and regulation. Antioxid. Redox. Signal.

[20] Lee W., Probst S., Santoyo-Sánchez M., Al-Hamdani W., Diebels I., von Sivers J., Kerek E., Prenner E., Thévenod F. (2017). Initial autophagic protection switches to disruption of autophagic flux by lysosomal instability during cadmium stress accrual in renal NRK-52E cells. Arch. Toxikol..

[21] Wang X., Yang H., Wang M., Yang D., Wang Z., Wang L. (2017). Trehalose protects against cadmium-induced cytotoxicity in primary rat proximal tubular cells via inhibiting apoptosis and restoring autophagic flux. Cell Death Dis..

[22] Chen S., Liu G., Long M., Zou H., Cui H. (2018). Alpha lipoic acid attenuates cadmium-induced nephrotoxicity via the mitochondrial apoptotic pathways in rat. J. Inorg. Biochem..

[23] Wang H., Zhai N., Chen Y., Xu H., Huang K. (2017). Cadmium induces Ca mediated, calpain-1/caspase-3-dependent apoptosis in primary cultured rat proximal tubular cells. J. Inorg. Biochem..

[24] Liu W., Gong Z., Zhang K., Dong W., Zou H., Song R., Bian J., Zhu J., Liu G., Liu Z. (2022). Paeonol protects renal tubular cells against cadmium-induced cytotoxicity via alleviating oxidative stress, inhibiting inflammatory responses and restoring autophagy. J. Inorg. Biochem..

[25] Shi L., Cao H., Luo J., Liu P., Wang T., Hu G., Zhang C. (2017). Effects of molybdenum and cadmium on the oxidative damage and kidney apoptosis in Duck. Ecotoxicol Environ. Saf..

[26] Wang C., Nie G., Yang F., Chen J., Zhuang Y., Dai X., Liao Z., Yang Z., Cao H., Xing C. (2020). Molybdenum and cadmium co-induce oxidative stress and apoptosis through mitochondria-mediated pathway in duck renal tubular epithelial cells. J. Hazard. Mater..

[27] Haynes C., Ron D. (2010). The mitochondrial UPR—protecting organelle protein homeostasis. J. Cell Sci..

[28] Naresh N.U., Haynes C.M. (2019). Signaling and Regulation of the Mitochondrial Unfolded Protein Response. Cold Spring Harb. Perspect. Biol..

[29] Papa L., Germain D. (2014). SirT3 regulates the mitochondrial unfolded protein response. Mol. Cell. Biol..

[30] Shi Y., Dierckx A., Wanrooij P., Wanrooij S., Larsson N., Wilhelmsson L., Falkenberg M., Gustafsson C. (2012). Mammalian transcription factor A is a core component of the mitochondrial transcription machinery. Proc. Natl. Acad. Sci. USA.

[31] Dhar S., Ongwijitwat S. (2008). Wong-Riley, Nuclear respiratory factor 1 regulates all ten nuclear-encoded subunits of cytochrome c oxidase in neurons. J. Biol. Chem..

[32] Rauf A., Olatunde A., Imran M., Alhumaydhi F., Aljohani A., Khan S., Uddin M., Mitra S., Emran T., Khayrullin M. (2021). Honokiol: A review of its pharmacological potential and therapeutic insights. Phytomedicine Int. J. Phytother. Phytopharm..

[33] Sarrica A., Kirika N., Romeo M., Salmona M., Diomede L. (2018). Safety and Toxicology of Magnolol and Honokiol. Planta Med..

[34] Liu H., Wang T., Hsu Y., Chou C., Huang K., Hsu C., Liang H., Chang H., Lee T., Tsai P. (2019). Nanoparticulated Honokiol Mitigates Cisplatin-Induced Chronic Kidney Injury by Maintaining Mitochondria Antioxidant Capacity and Reducing Caspase 3-Associated Cellular Apoptosis. Antioxidants.

[35] Locatelli M., Zoja C., Zanchi C., Corna D., Villa S., Bolognini S., Novelli R., Perico L., Remuzzi G., Benigni A. (2020). Manipulating Sirtuin 3 pathway ameliorates renal damage in experimental diabetes. Sci. Rep..

[36] Yu Y., Li M., Su N., Zhang Z., Zhao H., Yu H., Xu Y. (2016). Honokiol protects against renal ischemia/reperfusion injury via the suppression of oxidative stress, iNOS, inflammation and STAT3 in rats. Mol. Med. Rep..

[37] Park E., Dusabimana T., Je J., Jeong K., Yun S., Kim H., Kim H., Park S. (2020). Honokiol Protects the Kidney from Renal Ischemia and Reperfusion Injury by Upregulating the Glutathione Biosynthetic Enzymes. Biomedicines.

[38] Thévenod F., Lee W. (2015). Live and Let Die: Roles of Autophagy in Cadmium Nephrotoxicity. Toxics.

[39] Omidifar N., Nili-Ahmadabadi A., Nakhostin-Ansari A., Lankarani K., Moghadami M., Mousavi S., Hashemi S., Gholami A., Shokripour M., Ebrahimi Z. (2021). The modulatory potential of herbal antioxidants against oxidative stress and heavy metal pollution: Plants against environmental oxidative stress. Environ. Sci. Pollut. Res. Int..

[40] Johri N., Jacquillet G., Unwin R. (2010). Heavy metal poisoning: The effects of cadmium on the kidney. Biometals.

[41] Sant’Ana M., Moraes R., Bernardi M. (2005). Toxicity of cadmium in Japanese quail: Evaluation of body weight, hepatic and renal function, and cellular immune response. Environ. Res..

[42] Li X., Zheng Y., Zhang G., Wang R., Jiang J., Zhao H. (2021). Cadmium induced cardiac toxicology in developing Japanese quail (*Coturnix japonica*): Histopathological damages, oxidative stress and myocardial muscle fiber formation disorder. Comp. Biochem. Physiol. C Toxicol. Pharm..

[43] Zhu Q., Li W., Zheng J. (2018). Life-cycle exposure to cadmium induced compensatory responses towards oxidative stress in the liver of female zebrafish. Chemosphere.

[44] Xia S., Lin H., Liu H., Lu Z., Wang H., Fan S., Li N. (2019). Honokiol Attenuates Sepsis-Associated Acute Kidney Injury via the Inhibition of Oxidative Stress and Inflammation. Inflammation.

[45] Wang T., Liu H., Lai Y., Jan T., Nomura N., Chang H., Chou C., Lee Y., Tsai P. (2018). Honokiol, a Polyphenol Natural Compound, Attenuates Cisplatin-Induced Acute Cytotoxicity in Renal Epithelial Cells Through Cellular Oxidative Stress and Cytoskeleton Modulations. Front. Pharmacol..

[46] Wang C., Nie G., Zhuang Y., Hu R., Wu H., Xing C., Li G., Hu G., Yang F., Zhang C. (2020). Inhibition of autophagy enhances cadmium-induced apoptosis in duck renal tubular epithelial cells. Ecotoxicol. Environ. Saf..

[47] Shen Z., Liu P., Sun Q., Li Y., Acharya R., Li X., Sun C. (2021). FTO inhibits UPR-induced apoptosis by activating JAK2/STAT3 pathway and reducing m6A level in adipocytes. Apoptosis.

[48] Wang Y., Jasper H., Toan S., Muid D., Chang X., Zhou H. (2021). Mitophagy coordinates the mitochondrial unfolded protein response to attenuate inflammation-mediated myocardial injury. Redox. Biol..

[49] Quiles J., Gustafsson Å. (2020). Mitochondrial Quality Control and Cellular Proteostasis: Two Sides of the Same Coin. Front. Physiol..

