# Supplementary material for: Honokiol Antagonizes Cadmium-Induced Nephrotoxicity in Quail by Alleviating Autophagy Dysfunction, Apoptosis and Mitochondrial UPR Inhibition with Its Antioxidant Properties
Source: Life (Basel). 2022 Oct 10;12(10):1574. doi: 10.3390/life12101574 (PMC9604973; doi:10.3390/life12101574)
Supplement: Supplementary file 1 [file life-12-01574-s001.zip › life-1938257-supplementary.pdf]

Table S1 Primer sequences of mRNA for QRT-PCR

Gene	Primer (5’→3’)	Product length/bp	Genbank No
Nrf2-F	AAACAGCGGCGGAACGAG	195	NC_029522.1
Nrf2-R	AAAAACTTCACGCCTTGCCC		
Hmox1-F	TCCTGGCACTCGGTTTCATC	159	NC_029516.1
Hmox1-R	ATCACAGCACCCAGTCGAAG		
Gss-F	ATGACGTGGACTGCCTTCTG	102	NC_029535.1
Gss-R	CCACATGGTTCTGGGCATCT		
Gpx4-F	TGATGCTCCCCTTCATCTCG	189	NC_029543.1
Gpx4-R	TCCAGCAGATGCACGCTAAA		
Sod2-F	GTTACAGCTCAGGTGTCGCT	155	NC_029518.1
Sod2-R	GCGAAAGAACCGAAGTCACG		
Lc3b-F	CGCACCTTCGAGCAAAGA	150	NC_029526.1
Lc3b-R	CATGTTGACGTGATCTGGCA		
Becn1-F	AACTGAAAGAACTGGCACTGGA	147	NC_029542.1
Becn1-R	GACCACTGTGCTGAGCTTCCT		
Atg5-F	GAACCGATTCCGGAGCTGAA	193	NC_029518.1
Atg5-R	TGAAGCAGGTTGGTATGCGT		
Sqstm1-F	CGCTGCTGGAGGTATTGAAGT	112	NC_029528.1
Sqstm1-R	AGTACTGCTGCTTGACTCGG		
Lamp2-F	GCTCGGAACCTGCGGTTTTT	197	NC_029519.1
Lamp2-R	TGTCACTGCCACAGATGCTT		
Tfeb-F	CTGGGCCTTCCAGTTTGGTT	127	NC_029541.1
Tfeb-R	TTGAAGCGAGACCTCCGTTG		
Rab7-F	GTGGTTGGGCCCGATAAAG	100	NC_029527.1
Rab7-R	GAAGTCATCTTTTAAACGTGGTGC		
Cstb-F	GAGGACAAGCACTACGGCAT	148	NC_029518.1
Cstb-R	CATGCTGGTACACCCCAGAT		

Gene	Primer (5'→3')	Product length/bp	Genbank No
Cstd-F	CACTGTCACCTGCTGGACAT	160	NC_029520.1
Cstd-R	TCAGGTTACCAAGCGTGACT		
Casp3-F	CCGGAGGTGAGGAGCTGAT	144	NC_029519.1
Casp3-R	TCCAGAATCCACAGACTTGCT		
Bak1-F	ATCTTCCTGAGGTGCTTCGC	100	NC_029541.1
Bak1-R	GGCTCGGTCATCTACCACAG		
Bcl2-F	TCATTTTGGATCTGCCGTCG	161	NC_029517.1
Bcl2-R	CGATGCTTTGATGCTTCGGG		
Sirt1-F	TGACAGGAGCTGGGGTGT	197	NC_029521.1
Sirt1-R	AGATGGCTGGAATTGGCCTG		
Sirt3-F	AGGGCTCCTTCTACGTCTCT	165	NC_029520.1
Sirt3-R	GCCATAACGTCTCCCCTGAA		
Nrf1-F	ACCGTCCGAGTTCCTGGATA	172	NC_029516.1
Nrf1-R	CCTGATGTCTCGGATGAGGC		
Ppargc1a-F	CAGACCCAGGAAGAGCGTC	109	NC_029519.1
Ppargc1a-R	TGCACTCCTCGATTTCACCAA		
Ppargc1β-F	GGAGAAACCCCCTTTCCAGG	111	NC_029528.1
Ppargc1β-R	ACCTGAAGGTGCATCTGCTT		
Tfam-F	GCAGCTCGCTCAAGACGATA	108	NC_029521.1
Tfam-R	CGCTGCTCTCTGGAACGTAT		
Gapdh-F	TGTCTATAAAGGGCGGCGGA	128	NC_029516.1
Gapdh-R	CCAATACGGCCAAATCCGTT		
